# Test-retest reliability of a questionnaire to assess physical environmental factors pertaining to physical activity

**DOI:** 10.1186/1479-5868-2-7

**Published:** 2005-06-15

**Authors:** Kelly R Evenson, Aileen P McGinn

**Affiliations:** 1 Department of Epidemiology, School of Public Health, University of North Carolina-Chapel Hill, Chapel Hill, North Carolina, USA

**Keywords:** environment, exercise, leisure activities, questionnaires, reproducibility of results

## Abstract

**Background:**

Despite the documented benefits of physical activity, many adults do not obtain the recommended amounts. Barriers to physical activity occur at multiple levels, including at the individual, interpersonal, and environmental levels. Only until more recently has there been a concerted focus on how the physical environment might affect physical activity behavior. With this new area of study, self-report measures should be psychometrically tested before use in research studies. Therefore the objective of this study was to document the test-retest reliability of a questionnaire designed to assess physical environmental factors that might be associated with physical activity in a diverse adult population.

**Methods:**

Test and retest surveys were conducted over the telephone with 106 African American and White women and men living in either Forsyth County, North Carolina or Jackson, Mississippi. Reliability of self-reported environmental factors across four domains (e.g., access to facilities and destinations, functionality and safety, aesthetics, natural environment) was determined using intraclass correlation coefficients (ICC) overall and separately by gender and race.

**Results:**

Generally items displayed moderate and sometimes substantial reliability (ICC between 0.4 to 0.8), with a few differences by gender or race, across each of the domains.

**Conclusion:**

This study provides some psychometric evidence for the use of many of these questions in studies examining the effect of self-reported physical environmental measures on physical activity behaviors, among African American and White women and men.

## Background

Physical activity improves health and quality of life and reduces the risk for several leading causes of death [1]. Yet despite these documented benefits, many adults do not obtain the recommended amounts of physical activity [1]. Barriers to physical activity occur at multiple levels: individual, interpersonal, organizational, community, and public policy or society factors. These factors fit within the framework of the socioecologic model [2,3]. Several studies have reviewed the literature on correlates of physical activity among adults and each has shown that until more recently the focus has been on individual and interpersonal levels of this framework and not on the broader contextual measures [4-7].

In a 1998 review, Sallis et al [8] recommended pursuing a range of strategies to improve the conceptualization of the environment for physical activity, identifying behavior settings in which people are most likely to be physically active, and identifying characteristics of settings that appear to decrease or increase the likelihood of physical activity in that setting. Since that time the research in this field has proliferated. With a new area of study, self-reported measures are needed that have been tested psychometrically on diverse populations, including assessment of reliability and validity if appropriate.

Pikora et al [9] developed a framework for assessing potential environmental influences of walking and cycling based on a review of the literature, interviews, and a Delphi study. The framework included the following physical environmental domains: destination, functionality, safety, and aesthetic. The destination feature relates to the availability of public and private facilities. The functionality feature reflects the physical attributes of the street and path that make up the fundamental structural aspects of the local environment, such as the type and width of the street and the volume, speed, and type of traffic. The safety feature represents both personal safety and traffic safety. The aesthetic feature included both streetscape (e.g., trees, garden and street maintenance, cleanliness, pollution) and views (e.g., sights, architecture).

Using the framework of Pikora et al [9], we developed survey questions or used other published questions to assess these features, since we hypothesized they might be associated with physical activity of adults. In addition, we also developed questions on the natural environment and the use of physical activity facilities. The purpose of this study was to evaluate the psychometric properties of the survey by evaluating the test-retest reliability of the questionnaire in a diverse adult population. In addition, we explored whether reliability differed by race or gender, since we wanted to ensure that the questionnaire was reliable among African American and White women and men, the target for a survey we were conducting.

## Methods

### Sample

A telephone survey was conducted using a computer assisted telephone interview system (CATI) between January and July 2003 on a random sample of non-institutionalized adults 18 years or older residing in two regions: Forsyth County, North Carolina (NC) and the metropolitan statistical area (MSA) of Jackson, Mississippi (MS). Disproportionate sampling was used for Forsyth County in order to ensure representation for less urban areas outside of the Winston-Salem metropolitan area within the county. Respondents were randomly chosen in two stages: the first stage at the household level and the second stage at the individual level. Surveys were only conducted in English.

A sampling company (Genesys Marketing Systems Group) provided a listing of residential household phone numbers and Clearwater Research, Inc (Boise, Idaho) conducted the telephone surveys. They used Behavioral Risk Factor Surveillance System (BRFSS) telephone survey protocols [10] of up to 15 call attempts for each sampled phone number distributed across weekday, weeknight, and weekends. The average length of the telephone interview was 27 minutes.

### Reliability Interviews

Overall 1662 men and women completed the baseline survey. At the end of the interview, 1448 adults were asked if they would be willing to participate in a retest interview. The remaining 214 adults were not asked to participate in a retest interview, because the interview quota was complete. Among these 1448 adults, 76% (n = 1104) agreed to be called back for the retest survey. Reliability information was collected from a 6% (n = 106) purposeful sample of women and men, to ensure approximately equal numbers of participants from both sites, by gender, and by race. The mean time between interviews was 16.8 days (standard deviation 4.2, range 9–30 days). On average, the reliability survey was 5 minutes shorter than the original survey, mainly due to the exclusion of questions on the random selection process and their familiarity with the survey procedures. Each participant provided consent and the study was approved by the Institutional Review Board at the University of North Carolina. Participants were paid $5 for their participation for each survey they completed.

### Questionnaire

Most questions on perceived environmental factors were unique to this study, but developed based on work and existing questionnaires by others [11-15]. The questions we examined for test-retest reliability are listed in Appendix 1. While we were guided by the framework of Pikora et al [9], it should be noted that we did not develop questions to ascertain all elements of their framework. Furthermore, some of the questions may fall in more than one feature of their framework. For example, hills could be considered as part of the functional feature, but we chose to group it under the natural environment. It should also be noted that while the majority of the 51 questions under evaluation focused on perceived environmental factors including availability of facilities, there were five questions that focused on frequency of use (how often do you walk to destinations and how often do you use private facilities, public facilities, public schools, and places of worship for physical activity).

#### Access to Facilities and Destinations

We expanded the destination definition of Pikora et al [9] which related to the availability of facilities within one's neighborhood, to also include access to facilities within the home. In part, this was due to the fact that we were interested in all types of leisure activity as well as transportation activity, and not just walking and cycling. In our survey, destinations were assessed by asking whether participants had places within walking distance. Participants were also asked whether their neighborhoods had sidewalks, trails, or parks and playgrounds and whether lack of these facilities was a barrier to their activity. Other questions pertained to having places to exercise, having equipment or facilities at home, and the availability, use, and quality of private facilities, public facilities, and public schools. Participants were also asked about the use and quality of activity facilities at places of worship.

In addition to exploring item-by-item responses, we calculated an "availability of physical activity facilities index" by adding the responses to the availability of private recreational facilities, public recreational facilities, and public school facilities together, with assignments of excellent = 4, good = 3, fair = 2, poor = 1, or none = 0. The higher the score the more facilities were available to participants.

#### Functionality and Safety

In our questionnaire, we combined the functionality and safety features of the framework of Pikora et al [9]. Functionality included questions on heavy traffic, speeding cars, noise, and crosswalks and traffic signals. For safety, we asked questions relating to dogs, personal safety, and street lighting. A "crime safety index" was also collected, as developed by Saelens et al [15], using the 6 items found in Appendix 1 and assigning values 1 through 4 to the response options, ranging from strongly disagree to strongly agree, with the last 3 questions (items 4, 5, 6) reverse coded. The score was calculated by adding the 6 items together (with the lower number indicating more crime in the neighborhood) and taking the mean, such that the score ranged from 1 to 4.

#### Aesthetics and Natural Environment

For the aesthetic feature, we included items on trees, pollution, and trash, litter, or graffiti. Questions pertaining to the natural environment included items on hills and weather.

#### Physical Activity

Physical activity was assessed by asking if the adults had participated in any moderate or vigorous activity for at least 10 minutes at a time, using questions from the year 2001 BRFSS core module on physical activity [16]. If they responded "yes" to either question, then they were asked how many days per week did they engage in the activity for at least 10 minutes at a time and how much total time per day they spent doing these activities. We grouped participants into three levels based on current physical activity recommendations [17]: those who met recommendations (defined as being moderately active for at least 30 minutes for 5–7 days a week or vigorously active at least 20 minutes for 3–7 days a week), those who were insufficiently active (defined as some physical activity, but not enough to meet recommendations), and those who were inactive (not participating in any moderate or vigorous physical activities for at least 10 minutes at a time in a usual week).

#### Socio-demographics and Health

All respondents were asked questions regarding age, race, education, and employment. Employment was grouped into two categories: employed or not employed (out of work, homemaker, student, retired, or unable to work). General health was assessed by asking, "Would you say your general health is: excellent, good, average, fair, or poor?" Respondents were also asked, "Are you limited in doing any physical activity or exercise because of a disability or health problem?" If they answered "yes", the respondent was asked if this disability of health problem was mild, moderate, or severe.

### Statistical Analyses

Analyses were conducted overall and by gender and race. Intraclass correlation coefficients (ICC) were calculated to assess reliability, based on a one-way analysis of variance [18], along with 95% confidence intervals. The ICC was the proportion of total variance in the measure (subject variability and measurement error) that was due to the true differences between participants (subject variability). We also calculated overall kappa (2 level) and weighted kappa (> = 3 level) coefficients for categorical variables, which were similar to the ICC and thus are not reported. Although the ICC ranges from 0 to 1, in a few cases when the sample size was small, the lower confidence interval fell below 0 and is reported as such. As a rough guide, we followed the ratings suggested by Landis and Koch [19] an agreement level: 0–0.2 poor, 0.2–0.4 fair, 0.4–0.6 moderate, 0.6–0.8 substantial, and 0.8-<1.0 almost perfect. For the index measures, Cronbach alpha coefficients were calculated to assess internal consistency. SAS version 8.01 was used for all analyses.

## Results

Among the sample of 106 adults, approximately one-quarter were from each race-gender group: n = 27 African American women, n = 25 African American men, n = 30 White women, and n = 24 White men. Approximately half were from Forsyth County, NC and half were from Jackson, MS. The mean age of participants was 48 years (range 18 to 82 years). The mean and median length of time living at the residence was 13.4 and 7.4 years, respectively (interquartile range 1.8 to 23.0 years). Based on the self-reported data, 42.5% met recommendations for physical activity, 44.3% were insufficiently active, and 13.2% were inactive. Other descriptive characteristics are listed in Table 1 (Additional file: 1).

The test-retest reliability of all measures is reported overall (Table 2, Additional file: 1) and by gender and race (Table 3, Additional file: 1). When exploring differences in reliability by gender or race, we discuss here those measures where the ICC differed by at least two categories, according to Landis and Koch [19].

### Reliability of Items on Access to Facilities and Destinations

Items on general access and availability for places to exercise, home equipment and facilities, and neighborhood attributes of sidewalks, trails, and parks/playground (including whether these were a barrier to physical activity) showed moderate to substantial reliability, except for the lower reliability found on the item asking if lack of parks was a barrier to physical activity. Only two items meaningfully differed by gender (i.e., how often do you walk to those places and quality of worship facilities were both lower among women). Several items differed by race, with higher reliability found for African Americans on one question (i.e., having places to exercise) and lower reliability on three questions (i.e., how often using equipment at home, lack of sidewalks, lack of parks or playgrounds). The availability of physical activity facilities index had substantial test-retest agreement and the Cronbach alpha coefficient was 0.81. When examining component questions, the item on availability of public recreational facilities showed higher reliability among men. In general the questions on quality had lower reliability, which may be due in part to the difficult in assessing quality and due to the small sample size because of the skip patterns for those questions.

### Reliability of Items on Functionality and Safety

For functionality and safety, the items assessing whether characteristics were a problem in their neighborhood and whether those items were barriers to their physical activity showed moderate to substantial reliability. Only the questions on noise showed differences by gender and race. Reliability was higher among (1) Whites compared to African Americans when determining whether noise was a problem in their neighborhood and (2) among men compared to women when determining whether excessive noise was a barrier to physical activity. The crime safety index had substantial reliability overall, with one component item (i.e., if walkers and bikers on the streets can be seen) performing poorly. For Whites, the component question on talking with people when walking also performed poorly.

### Reliability of Items on Aesthetics

The six items pertaining to trash, trees, and pollution represented the aesthetics domain. These items generally were moderately reliable. Reliability was higher among men compared to women and among Whites compared to African Americans when assessing if lack of trees were a problem in their neighborhood. African Americans had higher reliability than White participants on the question pertaining to whether exhaust fumes was a barrier to their physical activity.

### Reliability of Items on Natural Environment

The four items pertaining to hills and weather represented the natural environment and generally showed moderate reliability. No meaningful differences were identified by gender and only one difference was found by race. Reliability was somewhat higher among African Americans when determining whether bad weather was a problem.

## Discussion

This study documents the test-retest reliability of a questionnaire designed to assess physical environmental factors that might be associated with physical activity in a diverse adult population. Many of the items and scales had moderate and sometimes substantial reliability (ICC between 0.4 to 0.8), with a few differences by gender or race, across each of the domains (e.g., awareness and access to facilities, functionality and safety, aesthetics, and natural environment).

### Awareness and Access to Facilities and Destinations

Within the domain of awareness and access to facilities and destinations, we evaluated 21 survey items. In a 2002 review, Humpel et al [20] concluded that most studies examining the relationship between self-reported accessibility to physical activity facilities and physical activity have shown a positive association. We developed a 3-item index to assess availability of physical activity facilities index, which demonstrated substantial reliability. These indices asked about private, public, and public school recreational facilities. Although we asked about use of physical activity facilities at places of worship, we did not inquire about availability of those facilities, and therefore did not include it in our index measures. Future studies may want to consider adding and testing the addition of this question to the index. In general, the questions on quality of these activity facilities had lower reliability, which may be due in part to the difficult in assessing quality and due to the small sample size because of the skip patterns for those questions.

Several studies have examined items on neighborhood features hypothesized to be correlated with physical activity, as first used by Sallis et al [11], to determine presence or absence of characteristics such as sidewalks, hills, heavy traffic, street lights, and unattended dogs. We chose to expand this concept, to ask whether the respondent agreed or disagreed that lack of these items were a problem in their neighborhood, and whether those items were a barrier to their physical activity. The items on availability and barriers to physical activity pertaining to sidewalks, trails, and parks generally displayed acceptable reliability. The question on places to go within walking distance came from another survey [14]. In another study of 344 multi-ethnic women, this question showed substantial reliability (ICC 0.75) [21], which was similar to our findings (ICC 0.63 overall, ICC 0.57 for women).

### Functionality and Safety

Functionality refers to design features of the built environment, such as noise, traffic, speeding cars, and crosswalks [9]. Increased functionality and safety are hypothesized to be associated with participation in physical activity [20,22]. Most of the items we examined in this domain were newly derived and displayed acceptable reliability. The crime safety index, which came from Saelens et al [15], had substantial reliability in this study (ICC 0.68). In Saelens et al reliability study of a similar sample size, the ICC for the crime safety index was a bit higher, with an ICC of 0.80; however, these estimates both fall within the same broad category as being "substantial" in reliability.

### Aesthetics and Natural Environment

Aesthetic features, such as trees, pollution, and trash, can be difficult to measure but are likely associated with whether or not an individual chooses to be active outdoors [20]. The natural environment, such as weather and hills, are also hypothesized to be associated with physical activity. The survey items for the natural environment and most items for aesthetics were newly derived and displayed moderate reliability.

### Implications

These self-reported measures can be used to document individual level perceptions of the neighborhood environment and to explore their association with physical activity. There is also interest in studying the level of agreement between these neighborhood perceptions as compared to objective measures of the neighborhood environment, and the strength of their relationships with physical activity [23,24], which have not been adequately explored in the literature. In some cases, if studies find that perceived measures do not adequately represent a similar objectively measured construct, then this could point to interventions. For example, if an individual perceives that there are no trails in their neighborhood, but the objective measure indicates that there is one nearby, this could indicate the need for better promotion of existing resources. These self-reported measures can also be used to aggregate up responses to create a neighborhood level measure.

Future studies can also utilize these questions to further explore the mediators of change in physical activity. For example, it may be that the perception of an environmental factor as a barrier to physical activity could be a mediating variable (to be targeted in an intervention) between perceived environmental factors and physical activity behavior. This could not be tested within this cross-sectional study but could be explored in future studies.

### Study Limitations

Our study had several limitations. First, despite the short time between administrations, true changes, while unlikely, could have occurred between surveys, which would weaken the reported reliability estimates. Second, in the analysis we could not account for whether the same interviewer administered the test and retest interview, but all retests were conducted using similar standardized methodology. Third, the generalizability of this study is somewhat limited in that it was conducted in only two geographic areas and only among African American and White participants. Some of our reliability estimates, especially in the stratified analysis by gender and race, were not very precise due to the sample size. However, we were able to explore whether reliability of these questions differed by race or gender, providing estimates that might be more useful to studies focusing on certain race or gender groups. Future studies should consider their population under study, to determine whether these findings might be generalizable to their sample.

One challenge of this research is deciding if and how to define neighborhood. In this survey, we decided to define "neighborhood" for the participant as a 20 minute walk or one mile from their home. However, for the availability of private facilities, public facilities, public schools, and places of worship, we defined the "community" as the area within a 20-minute drive from their home. In doing this, it may have presented challenges to the participant about recalling geographic areas that may not be familiar to them. There may also have been other factors that affected recall, and thus reliability results, such as disability and the length of time living in the neighborhood. In this study, only 19% of participants reported a moderate or severe disability that might limit their physical activity and 75% of participants reported living at their address 1.8 years or longer. Thus, these were stratification factors that could not be adequately explored with this data.

## Conclusion

In conclusion, this study provides some psychometric evidence for the use of many of these environmental measures in future studies examining the effect of self-reported environmental measures on physical activity behaviors for African American and White women and men. The evidence for test-retest reliability of the questionnaire is especially important, as work in this field is expanding rapidly. To date, one study has compared objective to self-reported estimates of several physical environmental measures we used here (e.g., traffic, sidewalks, lights, and crime) [24]. Further work in this area will help to better understand which measures to use and how to interpret findings from these measures.

## Competing interests

The author(s) declare that they have no competing interests.

## Authors' contributions

KRE and APM conducted the study, KRE drafted the manuscript, and APM assisted with the analysis and interpretation. Both authors read and approved the final manuscript.

## Supplementary Material

Additional file 1 Click here for file
