# Supplementary material for: Test-retest reliability of a questionnaire to assess physical environmental factors pertaining to physical activity
Source: Int J Behav Nutr Phys Act. 2005 Jun 15;2:7. doi: 10.1186/1479-5868-2-7 (PMC1183237; doi:10.1186/1479-5868-2-7)
Supplement: Additional file 1 [file 1479-5868-2-7-S1.doc]

Appendix 1: Physical environmental questions evaluated for test-retest reliability

| **Question** | **Response Options** |
| --- | --- |
| **Access to Facilities and Destinations** |  |
| Do you have places to be physically active? | Yes, no |
| *Are these places indoor, outdoor, or both? | Indoor only, outdoor only, both |
| Do you have any equipment or facilities at home for physical activity, such as an exercise bike, gym equipment, weights, a swimming pool, or exercise videos? | Yes, no |
| *How often do you use any of these pieces of equipment or facilities at home? Would you say … | Very often, often, sometimes, never |
| In your community, are there businesses or places where you need to go, such as stores or churches, where you can walk instead of drive? (14) | Yes, no |
| *How often do you walk there? | Very often, often, sometimes, never |
| No sidewalks or poorly maintained sidewalks are a problem in my neighborhood. Do you … | Strongly agree, agree, disagree, strongly disagree |
| Please indicate whether any of the following barriers keep you from any outdoor physical activities, such as walking, biking, or gardening in your neighborhood. Again think of your neighborhood as the area within about a 20 minute walk or one mile from your home as you think about your answers to these questions.  Is the lack of sidewalks or poorly maintained sidewalks a barrier? | Yes, no |
| Lack of walking, jogging, or biking trails is a problem in my neighborhood. Do you … | Strongly agree, agree, disagree, strongly disagree |
| Is the lack of walking, jogging, or biking trails a barrier? | Yes, no |
| Lack of parks or playgrounds is a problem in my neighborhood. Do you … | Strongly agree, agree, disagree, strongly disagree |
| Is the lack of parks or playgrounds a barrier?** | Yes, no |
| The next few questions are about facilities that may be available to you in your community. …. Please think of your community as the area within a 20 minute drive from your home. Private recreational facilities are places to be physically active, which you have to join or pay a fee to use. Examples of private facilities include YMCA’s, health clubs or gyms, martial arts studios, dance studios, or yoga studios. Would you say that the availability of private recreational facilities in your community was …. | Excellent, good, fair, poor, there are no private facilities in my community |
| *How often do you use the private recreational facilities in your local area? Would you say … | Very often, often, sometimes, never |
| *Would you say that the quality of the private recreational facilities in your local area was … | Excellent, good, fair, poor |
| The next few questions are about the public recreational facilities in your community or public places where people can walk to get exercise that are free and open to the public but are not located in public schools. Examples of public facilities are playgrounds, public pools, or community centers. Would you say that the availability of public recreational facilities in your community was … | Excellent, good, fair, poor, there are no public facilities in my community |
| *How often do you use the public recreational facilities? Would you say … | Very often, often, sometimes, never |
| *Would you say that the quality of the public recreational facilities in your local area was … | Excellent, good, fair, poor |
| The next few questions are about physical activity facilities that may be available at public schools in your community. These facilities may include fields, tracks, gyms, or swimming pools. Would you say the availability of facilities at public schools in your community was … | Excellent, good, fair, poor, there are no public schools available in my community |
| *How often do you use the public school facilities? Would you say … | Very often, often, sometimes, never |
| *Would you say that the quality of the public school recreational facilities was … | Excellent, good, fair, poor |
| How often do you use exercise facilities at a place of worship, such as a church? Would you say | Very often, often, sometimes, never, my church does not have exercise facilities |
| *Would you say the quality of these facilities was … | Excellent, good, fair, poor |
| Functionality and Safety |  |
| Excessive noise is a problem in my neighborhood. Do you … | Strongly agree, agree, disagree, strongly disagree |
| Is excessive noise a barrier? | Yes, no |
| Heavy traffic is a problem in my neighborhood. Do you … | Strongly agree, agree, disagree, strongly disagree |
| Is heavy traffic a barrier? | Yes, no |
| Speeding cars is a problem in my neighborhood. Do you … | Strongly agree, agree, disagree, strongly disagree |
| Are speeding cars a barrier? | Yes, no |
| Lack of crosswalks or traffic signals to help cross streets is a problem in my neighborhood. Do you … | Strongly agree, agree, disagree, strongly disagree |
| Is the lack of crosswalks or traffic signals a barrier? | Yes, no |
| Unattended dogs are a problem in my neighborhood. Do you … | Strongly agree, agree, disagree, strongly disagree |
| Are unattended dogs a barrier? | Yes, no |
| Is crime or fear for your personal safety a barrier? | Yes, no |
| Is inadequate street lighting a barrier? | Yes, no |
| Crime Safety Index (6 item) (15) |  |
| (1) My neighborhood streets are well lit. Do you … | Strongly agree, agree, disagree, strongly disagree |
| (2) Walkers and bikers on the street in my neighborhood can be easily seen by people in their homes. Do you … |
| (3) I see and speak to other people when I am walking in my neighborhood. Do you … |
| (4) There is a high crime rate in my neighborhood. Do you … |
| (5) The crime rate in my in my neighborhood makes it unsafe to go on walks during the day. Do you … |
| (6) The crime rate in my in my neighborhood makes it unsafe to go on walks at night. Do you … |
| Aesthetics |  |
| Trash, litter, or graffiti is a problem in my neighborhood. Do you … | Strongly agree, agree, disagree, strongly disagree |
| Are trash, litter, or graffiti barriers? | Yes, no |
| Lack of trees along the street that provide shade is a problem in my neighborhood. Do you … | Strongly agree, agree, disagree, strongly disagree |
| Is the lack of trees along the street that provides shade a barrier? | Yes, no |
| Exhaust fumes or other pollution is a problem in my neighborhood. Do you … | Strongly agree, agree, disagree, strongly disagree |
| Are exhaust fumes or other pollution a barrier? | Yes, no |
| Natural Environment |  |
| Hills or steep slopes are common in my neighborhood. Do you … | Strongly agree, agree, disagree, strongly disagree |
| Are hills or steep slopes a barrier? | Yes, no |
| In general, the weather is a problem in my neighborhood. Do you … | Strongly agree, agree, disagree, strongly disagree |
| Is weather a barrier? | Yes, no |

Items denoted with a * involved a skip pattern. If the participant answered no or no access to the prior question, then this question was no asked.

** The items on barriers to physical activity continue, starting with the question on noise.

Table 1. Baseline survey characteristics from participants completing reliability surveys (n=106)

| Factors | N | Percent |
| --- | --- | --- |
| Gender |  |  |
| Female | 57 | 57.8 |
| Male | 49 | 46.2 |
| Age (years) |  |  |
| 18-29 | 18 | 17.0 |
| 30-44 | 29 | 27.3 |
| 45-64 | 41 | 38.7 |
| 65+ | 18 | 17.0 |
| Education |  |  |
| Less than high school | 7 | 6.6 |
| High school graduate/GED | 29 | 27.4 |
| Some technical  school/college | 28 | 26.4 |
| College graduate | 42 | 39.6 |
| Employment |  |  |
| Employed | 61 | 58.1 |
| Not Employed | 44 | 41.9 |
| Race |  |  |
| White | 54 | 50.9 |
| African American | 52 | 49.1 |
| General Health |  |  |
| Excellent | 20 | 19.0 |
| Very good | 43 | 41.0 |
| Good | 21 | 20.0 |
| Fair/poor | 21 | 20.0 |
| Disability that Affects Physical Activity |  |  |
| None | 82 | 77.3 |
| Mild | 4 | 3.8 |
| Moderate | 13 | 12.3 |
| Severe | 7 | 6.6 |
| Physical Activity |  |  |
| Met recommendations | 45 | 42.5 |
| Insufficiently active | 47 | 44.3 |
| Inactive | 14 | 13.2 |
| Location |  |  |
| Forsyth County, North Carolina | 54 | 50.9 |
| Jackson, Mississippi | 52 | 49.1 |

Table 2. Sample proportions (from the first survey) and test-retest reliability using intraclass correlation coefficients (ICC) with 95% confidence intervals (CI), from the reliability survey (n=106)

|  | Response Options | Sample Proportions in % (n) | ICC  (n=106) | 95% CI |
| --- | --- | --- | --- | --- |
| Access to Facilities & Destinations |  |  |  |  |
| Have places to exercise | Indoor only | 9.5 (10) | 0.52 | (0.36, 0.64) |
|  | Outdoor only | 20.0 (21) |  |  |
|  | Indoor & outdoor | 62.9 (66) |  |  |
|  | None | 7.6 (8) |  |  |
| Having equipment/facilities at home | Yes | 56.6 (60) | 0.87 | (0.81, 0.91) |
|  | No | 43.4 (46) |  |  |
| *How often do you use equipment/facilities at home | Very often/Often | 15.0 (9) | 0.50 | (0.29, 0.67) |
|  | Sometimes/Never | 85.0 (51) |  |  |
| Places within walking distance | Yes | 68.9 (73) | 0.63 | (0.49, 0.73) |
|  | No | 31.1 (33) |  |  |
| *How often walking to those places | Very often/Often | 12.3 (9) | 0.80 | (0.71, 0.87) |
|  | Sometimes/Never | 87.7 (64) |  |  |
| Lack of sidewalks | Strongly agree/Agree | 50.5 (51) | 0.57 | (0.43, 0.69) |
|  | Disagree/Strongly disagree | 49.5 (50) |  |  |
| Lack of sidewalks is a barrier | Yes | 44.3 (47) | 0.49 | (0.33, 0.62) |
|  | No | 55.7 (59) |  |  |
| Lack of trail | Strongly agree/Agree | 42.5 (45) | 0.56 | (0.42, 0.68) |
|  | Disagree/Strongly disagree | 57.5 (61) |  |  |
| Lack of trail is a barrier | Yes | 36.6 (35) | 0.56 | (0.41, 0.68) |
|  | No | 66.4 (69) |  |  |
| Lack of parks/playground | Strongly agree/Agree | 29.3 (31) | 0.47 | (0.31, 0.61) |
|  | Disagree/Strongly disagree | 70.7 (75) |  |  |
| Lack of parks is a barrier | Yes | 27.4 (29) | 0.32 | (0.14, 0.48) |
|  | No | 72.6 (77) |  |  |
| Availability of physical activity facilities index | Score |  | 0.67 | (0.54, 0.76) |
| Availability of private recreational facilities | Excellent/Good | 86.4 (89) | 0.60 | (0.46, 0.71) |
|  | Fair/Poor/None | 15.6 (14) |  |  |
| *How often do you use private recreational facilities | Very often/often | 22.3 (23) | 0.68 | (0.56, 0.77) |
|  | Sometimes/never | 77.7 (80) |  |  |
| *Quality of private recreational facilities | Excellent/Good | 89.7 (35) | 0.37 | (0.10, 0.59) |
|  | Fair/Poor | 10.3 (4) |  |  |
| Availability of public recreational facilities | Excellent/Good | 67.6 (69) | 0.51 | (0.36, 0.64) |
|  | Fair/Poor/None | 32.4 (33) |  |  |
| *How often do you use public recreational facilities | Very often/often | 12.7 (13) | 0.55 | (0.40, 0.67) |
|  | Sometimes/never | 87.3 (89) |  |  |
| *Quality of public recreational facilities | Excellent/Good | 77.8 (42) | 0.43 | (0.22, 0.60) |
|  | Fair/Poor | 22.2 (12) |  |  |
| Availability of public school recreational facilities | Excellent/Good | 58.2 (53) | 0.39 | (0.21, 0.55) |
|  | Fair/Poor/None | 41.8 (38) |  |  |
| *How often do you use public school recreational facilities | Very often/often | 4.4 (4) | 0.51 | (0.35, 0.64) |
|  | Sometimes/never | 95.6 (86) |  |  |
| *Quality of public school recreational facilities | Excellent/Good | 73.1 (19) | 0.16 | (-0.18, 0.46) |
|  | Fair/Poor | 26.9 (7) |  |  |
| How often do you use places of worship | Very often/often | 8.5 (9) | 0.65 | (0.53, 0.75) |
|  | Sometimes/never/not available | 91.5 (97) |  |  |
| *Quality of those facilities at places of worship | Excellent/Good | 68.2 (15) | 0.50 | (0.20, 0.72) |
|  | Fair/Poor | 31.8 (7) |  |  |
|  |  |  |  |  |
| Functionality and Safety |  |  |  |  |
| Noise | Strongly agree/Agree | 24.5 (26) | 0.48 | (0.32, 0.62) |
|  | Disagree/Strongly disagree | 75.5 (80) |  |  |
| Excessive noise is a barrier | Yes | 11.3 (12) | 0.67 | (0.55, 0.77) |
|  | No | 88.7 (94) |  |  |
| Heavy traffic | Strongly agree/Agree | 35.9 (38) | 0.67 | (0.54, 0.76) |
|  | Disagree/Strongly disagree | 64.2 (68) |  |  |
| Heavy traffic is a barrier | Yes | 28.3 (30) | 0.67 | (0.55, 0.76) |
|  | No | 71.7 (76) |  |  |
| Speeding cars | Strongly agree/Agree | 41.5 (44) | 0.65 | (0.52, 0.74) |
|  | Disagree/Strongly disagree | 58.5 (62) |  |  |
| Speeding cars are barrier | Yes | 34.0 (36) | 0.67 | (0.55, 0.76) |
|  | No | 66.0 (70) |  |  |
| Lack of crosswalks or traffic signals | Strongly agree/Agree | 30.5 (32) | 0.45 | (0.29, 0.59) |
|  | Disagree/Strongly disagree | 69.5 (73) |  |  |
| Lack of crosswalks or traffic signals is a barrier | Yes | 28.3 (30) | 0.51 | (0.36, 0.64) |
|  | No | 71.7 (76) |  |  |
| Unattended dogs | Strongly agree/Agree | 33.0 (35) | 0.63 | (0.50, 0.73) |
|  | Disagree/Strongly disagree | 67.0 (71) |  |  |
| Unattended dogs are barrier | Yes | 26.4 (28) | 0.63 | (0.49, 0.73) |
|  | No | 73.6 (78) |  |  |
| Crime is a barrier | Yes | 25.5 (27) | 0.79 | (0.71, 0.85) |
|  | No | 74.5 (79) |  |  |
| Inadequate street lighting is a barrier | Yes | 24.8 (26) | 0.60 | (0.47, 0.71) |
|  | No | 75.2 (79) |  |  |
| Crime safety index | Score |  | 0.68 | (0.57, 0.77) |
| (1) If street is well lit | Strongly agree/Agree | 66.0 (70) | 0.46 | (0.29, 0.60) |
|  | Disagree/Strongly disagree | 34.0 (36) |  |  |
| (2) If walkers/bikers on the streets can be seen | Strongly agree/Agree | 76.4 (81) | 0.19 | (0.00, 0.37) |
|  | Disagree/Strongly disagree | 23.6 (25) |  |  |
| (3) If talking with people when walking | Strongly agree/Agree | 83.5 (86) | 0.28 | (0.09, 0.44) |
|  | Disagree/Strongly disagree | 16.5 (17) |  |  |
| (4) There is a high crime rate | Strongly agree/Agree | 29.3 (31) | 0.65 | (0.53, 0.75) |
|  | Disagree/Strongly disagree | 70.7 (75) |  |  |
| (5) Crime rate makes it unsafe for walking in the day | Strongly agree/Agree | 7.6 (8) | 0.49 | (0.33, 0.62) |
|  | Disagree/Strongly disagree | 92.4 (98) |  |  |
| (6) Crime rate makes it unsafe for walking at night | Strongly agree/Agree | 41.0 (43) | 0.73 | (0.63, 0.81) |
|  | Disagree/Strongly disagree | 59.0 (62) |  |  |
|  |  |  |  |  |
| Aesthetics |  |  |  |  |
| Trash | Strongly agree/Agree | 22.6 (24) | 0.64 | (0.52, 0.74) |
|  | Disagree/Strongly disagree | 77.4 (82) |  |  |
| Trash is a barrier | Yes | 9.4 (10) | 0.37 | (0.19, 0.52) |
|  | No | 90.6 (96) |  |  |
| Lack of trees along the street | Strongly agree/Agree | 22.6 (24) | 0.54 | (0.39, 0.66) |
|  | Disagree/Strongly disagree | 77.4 (82) |  |  |
| Lack of trees along the street is a barrier | Yes | 15.2 (16) | 0.39 | (0.22, 0.54) |
|  | No | 84.8 (89) |  |  |
| Exhaust fumes (pollution) | Strongly agree/Agree | 17.0 (18) | 0.50 | (0.35, 0.63) |
|  | Disagree/Strongly disagree | 83.0 (88) |  |  |
| Exhaust fumes (pollution) is a barrier | Yes | 12.3 (13) | 0.47 | (0.31, 0.61) |
|  | No | 87.7 (93) |  |  |
|  |  |  |  |  |
| Natural Environment |  |  |  |  |
| If hills or steep slopes are common | Strongly agree/Agree | 34.0 (36) | 0.53 | (0.38, 0.66) |
|  | Disagree/Strongly disagree | 66.0 (70) |  |  |
| Hills or steep slopes is a barrier | Yes | 10.4 (11) | 0.60 | (0.46, 0.71) |
|  | No | 89.6 (95) |  |  |
| Bad weather | Strongly agree/Agree | 25.0 (26) | 0.34 | (0.16, 0.50) |
|  | Disagree/Strongly disagree | 75.0 (78) |  |  |
| Weather is a barrier | Yes | 20.0 (21) | 0.56 | (0.42, 0.68) |
|  | No | 80.0 (84) |  |  |

Note: Although the sample proportions are categorized in the table, they were not categorized to calculate the ICC's.

The sample sizes may not add to n=106 due to missing values or due to a skip pattern (denoted with a *).

Table 3. Test-retest reliability using intraclass correlation coefficients (ICC) with 95% confidence intervals (CI) by race and gender, from the reliability survey (n=106)

|  | Gender | | | | Race | | | |
| --- | --- | --- | --- | --- | --- | --- | --- | --- |
|  | Male  (n=49) | | Female  (n=57) | | White  (n=54) | | African American  (n=52) | |
|  |
|  | ICC | 95% CI | ICC | 95% CI | ICC | 95% CI | ICC | 95% CI |
| Access to Facilities & Destinations |  |  |  |  |  |  |  |  |
| Have places to exercise | 0.42 | (0.16, 0.62) | 0.61 | (0.41, 0.75) | 0.35 | (0.09, 0.56) | 0.66 | (0.48, 0.79) |
| Having equipment/facilities at home | 0.79 | (0.66, 0.88) | 0.93 | (0.89, 0.96) | 0.96 | (0.93, 0.98) | 0.77 | (0.63, 0.86) |
| *How often do you use equipment/facilities at home | 0.50 | (0.20, 0.72) | 0.49 | (0.17, 0.72) | 0.66 | (0.44, 0.81) | 0.25 | (-0.14, 0.58) |
| Places within walking distance | 0.69 | (0.50, 0.81) | 0.57 | (0.37, 0.72) | 0.62 | (0.43, 0.76) | 0.59 | (0.38, 0.74) |
| *How often walking to those places | 0.92 | (0.86, 0.96) | 0.50 | (0.25, 0.69) | 0.68 | (0.46, 0.82) | 0.84 | (0.73, 0.91) |
| Lack of sidewalks | 0.67 | (0.49, 0.80) | 0.50 | (0.27, 0.67) | 0.74 | (0.59, 0.84) | 0.38 | (0.12, 0.59) |
| Lack of sidewalks is a barrier | 0.51 | (0.27, 0.69) | 0.48 | (0.25, 0.66) | 0.53 | (0.30, 0.69) | 0.45 | (0.20, 0.64) |
| Lack of trail | 0.67 | (0.48, 0.80) | 0.46 | (0.23, 0.64) | 0.65 | (0.46, 0.78) | 0.48 | (0.24, 0.66) |
| Lack of trail is a barrier | 0.63 | (0.43, 0.77) | 0.50 | (0.28, 0.67) | 0.73 | (0.58, 0.83) | 0.40 | (0.14, 0.60) |
| Lack of parks/playground | 0.52 | (0.28, 0.69) | 0.45 | (0.21, 0.63) | 0.66 | (0.47, 0.78) | 0.29 | (0.03, 0.52) |
| Lack of parks is a barrier | 0.37 | (0.11, 0.59) | 0.25 | (-0.01, 0.48) | 0.29 | (0.03, 0.52) | 0.36 | (0.10, 0.57) |
| Availability of physical activity facilities index | 0.69 | (0.50, 0.81) | 0.65 | (0.46, 0.78) | 0.54 | (0.30, 0.71) | 0.75 | (0.59, 0.85) |
| Availability of private recreational facilities | 0.54 | (0.31, 0.71) | 0.67 | (0.49, 0.79) | 0.58 | (0.37, 0.73) | 0.57 | (0.35, 0.73) |
| *How often do you use private recreational facilities | 0.74 | (0.59, 0.85) | 0.61 | (0.41, 0.75) | 0.68 | (0.50, 0.80) | 0.68 | (0.51, 0.81) |
| *Quality of private recreational facilities | 0.39 | (-0.01, 0.69) | 0.34 | (-0.05, 0.64) | 0.41 | (0.03, 0.69) | 0.29 | (-0.12, 0.62) |
| Availability of public recreational facilities | 0.64 | (0.43, 0.78) | 0.36 | (0.11, 0.56) | 0.48 | (0.24, 0.66) | 0.55 | (0.33, 0.71) |
| *How often do you use public recreational facilities | 0.57 | (0.34, 0.73) | 0.52 | (0.30, 0.68) | 0.50 | (0.27, 0.68) | 0.58 | (0.36, 0.73) |
| *Quality of public recreational facilities | 0.36 | (0.01, 0.62) | 0.48 | (0.19, 0.69) | 0.38 | (0.06, 0.64) | 0.47 | (0.18, 0.69) |
| Availability of public school recreational facilities | 0.23 | (-0.06, 0.49) | 0.53 | (0.31, 0.70) | 0.40 | (0.13, 0.61) | 0.37 | (0.10, 0.58) |
| *How often do you use public school recreational facilities | 0.44 | (0.17, 0.64) | 0.60 | (0.40, 0.75) | 0.35 | (0.07, 0.57) | 0.58 | (0.36, 0.74) |
| *Quality of public school recreational facilities | 0.14 | (-0.31, 0.55) | 0.17 | (-0.34, 0.61) | 0.19 | (-0.36, 0.66) | 0.10 | (-0.33, 0.50) |
| How often do you use places of worship | 0.71 | (0.54, 0.83) | 0.57 | (0.38, 0.72) | 0.60 | (0.40, 0.75) | 0.69 | (0.51, 0.81) |
| *Quality of those facilities at places of worship | 0.66 | (0.31, 0.87) | 0.27 | (-0.27, 0.69) | 0.48 | (-0.05, 0.80) | 0.53 | (0.12, 0.79) |
|  |  |  |  |  |  |  |  |  |
| Functionality and Safety |  |  |  |  |  |  |  |  |
| Noise | 0.38 | (0.12, 0.60) | 0.58 | (0.38, 0.73) | 0.69 | (0.52, 0.80) | 0.20 | (-0.08, 0.44) |
| Excessive noise is a barrier | 0.84 | (0.73, 0.90) | 0.52 | (0.30, 0.68) | 0.64 | (0.45, 0.77) | 0.68 | (0.51, 0.80) |
| Heavy traffic | 0.73 | (0.57, 0.84) | 0.61 | (0.42, 0.75) | 0.79 | (0.67, 0.87) | 0.52 | (0.29, 0.69) |
| Heavy traffic is a barrier | 0.54 | (0.31, 0.71) | 0.76 | (0.63, 0.85) | 0.72 | (0.56, 0.82) | 0.63 | (0.44, 0.77) |
| Speeding cars | 0.67 | (0.48, 0.80) | 0.63 | (0.44, 0.76) | 0.76 | (0.62, 0.85) | 0.52 | (0.29, 0.69) |
| Speeding cars are barrier | 0.65 | (0.45, 0.78) | 0.69 | (0.52, 0.80) | 0.78 | (0.66, 0.87) | 0.56 | (0.34, 0.72) |
| Lack of crosswalks or traffic signals | 0.47 | (0.20, 0.65) | 0.45 | (0.22, 0.63) | 0.51 | (0.28, 0.68) | 0.40 | (0.15, 0.61) |
| Lack of crosswalks or traffic signals is a barrier | 0.51 | (0.27, 0.69) | 0.52 | (0.30, 0.68) | 0.55 | (0.33, 0.71) | 0.48 | (0.24, 0.66) |
| Unattended dogs | 0.79 | (0.65, 0.87) | 0.52 | (0.30, 0.68) | 0.77 | (0.64, 0.86) | 0.53 | (0.31, 0.70) |
| Unattended dogs are barrier | 0.72 | (0.55, 0.83) | 0.55 | (0.35, 0.71) | 0.61 | (0.41, 0.75) | 0.62 | (0.42, 0.76) |
| Crime is a barrier | 0.85 | (0.75, 0.91) | 0.75 | (0.61, 0.85) | 0.73 | (0.57, 0.83) | 0.85 | (0.76, 0.91) |
| Inadequate street lighting is a barrier | 0.61 | (0.40, 0.76) | 0.60 | (0.41, 0.75) | 0.66 | (0.47, 0.78) | 0.56 | (0.34, 0.72) |
| Crime safety index | 0.72 | (0.55, 0.83) | 0.65 | (0.48, 0.78) | 0.76 | (0.63, 0.88) | 0.59 | (0.39, 0.74) |
| (1) If street is well lit | 0.58 | (0.37, 0.74) | 0.35 | (0.10, 0.56) | 0.45 | (0.21, 0.64) | 0.47 | (0.23, 0.66) |
| (2) If walkers/bikers on the streets can be seen | 0.27 | (0.00, 0.51) | 0.07 | (-0.19, 0.32) | 0.31 | (0.05, 0.53) | 0.11 | (-0.16, 0.37) |
| (3) If talking with people when walking | 0.30 | (0.03, 0.53) | 0.25 | (-0.01, 0.47) | 0.02 | (-0.24, 0.29) | 0.51 | (0.28, 0.69) |
| (4) There is a high crime rate | 0.69 | (0.51, 0.81) | 0.64 | (0.45, 0.77) | 0.78 | (0.65, 0.87) | 0.49 | (0.26, 0.67) |
| (5) Crime rate makes it unsafe for walking in the day | 0.55 | (0.32, 0.72) | 0.44 | (0.21, 0.63) | 0.52 | (0.30, 0.69) | 0.44 | (0.19, 0.63) |
| (6) Crime rate makes it unsafe for walking at night | 0.72 | (0.55, 0.83) | 0.73 | (0.58, 0.83) | 0.78 | (0.65, 0.87) | 0.67 | (0.48, 0.79) |
|  |  |  |  |  |  |  |  |  |
| Aesthetics |  |  |  |  |  |  |  |  |
| Trash | 0.70 | (0.52, 0.82) | 0.58 | (0.38, 0.73) | 0.70 | (0.54, 0.81) | 0.55 | (0.33, 0.72) |
| Trash is a barrier | 0.34 | (0.07, 0.57) | 0.40 | (0.16, 0.60) | 0.54 | (0.33, 0.71) | 0.26 | (-0.001, 0.49) |
| Lack of trees along the street | 0.68 | (0.50, 0.80) | 0.39 | (0.15, 0.59) | 0.72 | (0.56, 0.83) | 0.35 | (0.08, 0.56) |
| Lack of trees along the street is a barrier | 0.46 | (0.21, 0.66) | 0.30 | (0.04, 0.51) | 0.59 | (0.38, 0.74) | 0.27 | (-0.003, 0.50) |
| Exhaust fumes (pollution) | 0.52 | (0.28, 0.70) | 0.50 | (0.27, 0.67) | 0.54 | (0.32, 0.70) | 0.45 | (0.21, 0.65) |
| Exhaust fumes (pollution) is a barrier | 0.44 | (0.18, 0.64) | 0.51 | (0.30, 0.68) | 0.29 | (0.03, 0.52) | 0.62 | (0.42, 0.76) |
|  |  |  |  |  |  |  |  |  |
| Natural Environment |  |  |  |  |  |  |  |  |
| If hills or steep slopes are common | 0.47 | (0.22, 0.66) | 0.59 | (0.39, 0.74) | 0.57 | (0.36, 0.73) | 0.47 | (0.23, 0.66) |
| Hills or steep slopes is a barrier | 0.64 | (0.44, 0.78) | 0.57 | (0.37, 0.72) | 0.57 | (0.36, 0.72) | 0.65 | (0.46, 0.78) |
| Bad weather | 0.33 | (0.06, 0.55) | 0.36 | (0.12, 0.57) | 0.17 | (-0.10, 0.42) | 0.47 | (0.22, 0.65) |
| Weather is a barrier | 0.70 | (0.53, 0.82) | 0.45 | (0.22, 0.64) | 0.61 | (0.42, 0.75) | 0.52 | (0.29, 0.69) |

Items denoted with a * involved a skip pattern.
